# Automated Medical Records Review for Mild Cognitive Impairment and Dementia

**DOI:** 10.21203/rs.3.rs-5046441/v1

**Published:** 2024-09-09

**Authors:** Ruoqi Wei

**Affiliations:** University of Florida

**Keywords:** electronic health records (EHR), Alzheimer’s disease, dementia, mild cognitive impairment

## Abstract

**Objectives:**

Unstructured and structured data in electronic health records (EHR) are a rich source of information for research and quality improvement studies. However, extracting accurate information from EHR is labor-intensive. Here we introduce an automated EHR phenotyping model to identify patients with Alzheimer’s Disease, related dementias (ADRD), or mild cognitive impairment (MCI).

**Methods:**

We assembled medical notes and associated International Classification of Diseases (ICD) codes and medication prescriptions from 3,626 outpatient adults from two hospitals seen between February 2015 and June 2022. Ground truth annotations regarding the presence vs. absence of a diagnosis of MCI or ADRD were determined through manual chart review. Indicators extracted from notes included the presence of keywords and phrases in unstructured clinical notes, prescriptions of medications associated with MCI/ADRD, and ICD codes associated with MCI/ADRD. We trained a regularized logistic regression model to predict the ground truth annotations. Model performance was evaluated using area under the receiver operating curve (AUROC), area under the precision-recall curve (AUPRC), accuracy, specificity, precision/positive predictive value, recall/sensitivity, and F1 score (harmonic mean of precision and recall).

**Results:**

Thirty percent of patients in the cohort carried diagnoses of MCI/ADRD based on manual review. When evaluated on a held-out test set, the best model using clinical notes, ICDs, and medications, achieved an AUROC of 0.98, an AUPRC of 0.98, an accuracy of 0.93, a sensitivity (recall) of 0.91, a specificity of 0.96, a precision of 0.96, and an F1 score of 0.93 The estimated overall accuracy for patients randomly selected from EHRs was 99.88%.

**Conclusion:**

Automated EHR phenotyping accurately identifies patients with MCI/ADRD based on clinical notes, ICD codes, and medication records. This approach holds potential for large-scale MCI/ADRD research utilizing EHR databases.

## Introduction

Globally, 12% to 18% of people aged 60 or older are living with mild cognitive impairment (MCI) ^1^, and 10% to 15% of individuals living with MCI develop dementia each year ^2^. About one-third of people living with MCI due to Alzheimer’s disease (AD) develop dementia within five years^3^. The number of people in the United States with AD dementia will increase dramatically in the next 30 years due to growth of the population over the age of 65 ^4^. Observational data from Electronic Health Records (EHRs) are an increasingly important resource for research on risk factors and potential interventions for MCI and AD ^5–7^. A key challenge in scaling up EHR-based research is the accurate phenotyping of patients with a diagnosis of MCI and ADRD. Many studies rely on billing codes (International Classification of Diseases: ICD-9, ICD-10) ^8,9^; however, these are often inaccurate^10^. Manual review of clinical notes is more accurate ^11–15^ but labor intensive and impossible to conduct at large scale ^16–18^.

Automated EHR phenotyping seeks to address these challenges by automatically extracting information from clinical notes and combining this with structured information (e.g., medication prescriptions and diagnostic billing codes) to infer information of interest ^19^. Here, we present a machine learning (ML)-based EHR phenotyping model to automate the process of chart review for MCI/ADRD. We demonstrate that our model combining information from clinical notes, ICD codes, and medications provides an accurate MCI/ADRD phenotyping and is thus suitable for large-scale EHR research.

## Materials And Methods

### study cohort

**Figure 1:**
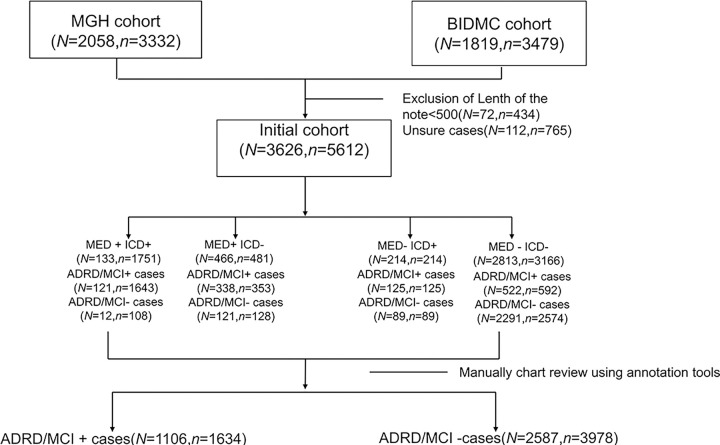
CONSORT diagram. *N* is the number of unique patients. *n* is the number of visits.

EHR data was extracted under protocols approved by the Massachusetts General Hospital (MGH) and Beth Israel Deaconess Medical Center (BIDMC) Institutional Review Boards with waivers of informed consent. A consort diagram is provided in Figure 1.

Patients were selected from MGH and BIDMC EHR archives from visits that took place between January 3^rd^, 2012, to November 3^rd^, 2017. Because ADRD/MCI is an age-associated disease, only patients aged 50 years or older were included. We randomly selected patients for inclusion using a stratified sampling strategy, to ensure adequate representation of patients with low, medium, and high likelihood of having an MCI/ADRD diagnosis to facilitate subsequent model development. Specifically, we created 4 groups based on the presence or absence of computable criteria (i.e. criteria that do not depend on analysis of unstructured text data): ‘MED−ICD−’, ‘MED+ICD+’, ‘MED+ICD−’, ‘MED−ICD+’, denoting groups of patients with and without ICD codes and medications (MED) associated with MCI/ADRD. Notes for patients within each of these groups were subsequently manually reviewed to determine which patients had an MCI/ADRD diagnosis (described below).

### study data

Study data included unstructured (i.e., free text) clinical notes and structured data. Structured data included International Classification of Diseases (ICD) codes ^20^ for MCI/ADRD, and dementia-related medications (see below). Clinical notes included office visit notes, admission notes, progress notes, discharge notes, and correspondence from all medical specialties in the MGH and BIDMC systems. Clinical notes contain a wide range of information such as chief complaint; history of present illness; physician examinations, observations, assessments, and treatment plans; active problems; and current and past medications. All patients included in the study had at least one clinical note. If a patient had any MED or ICD records, only notes recorded after the first appearance of a MED or ICD record were selected for analysis. We removed notes with fewer than 500 words as these were generally administrative notes without significant medical content.

### Ground truth labels: Manual chart review

We used a web-based tool developed in house that highlights keywords within notes from a provided list. A neurologist (MBW) performed manual review of all notes in each of the ‘MED+ ICD+’, ‘MED+ ICD−’, ‘MED− ICD+’, and ‘MED−ICD−’ groups, and assigned a final yes/no label regarding MCI/ADRD status. Cases marked as ‘uncertain’ were excluded.

In the ‘MED+ICD+’ group, patients were prescribed medications typically associated with ADRD/MCI (‘MED+’) and had ADRD/MCI-related ICD codes (‘ICD+’). In the ‘MED+ICD−’ group, patients were prescribed ADRD/MCI-related medication (‘MED+’), but no ADRD/MCI-related ICD codes (‘ICD−’) were present in their medical records. In the ‘MED− ICD+’ group, patients were not prescribed ADRD/MCI-related medications (‘MED−’), yet their medical records contained ADRD/MCI-related ICD codes (‘ICD+’). ‘MED−ICD−’ cases involved neither ADRD-related medication (‘MED−’) nor ADRD-related ICD codes (‘ICD−’) in the medical records.

### Predictors included in the model

**Figure 2:**
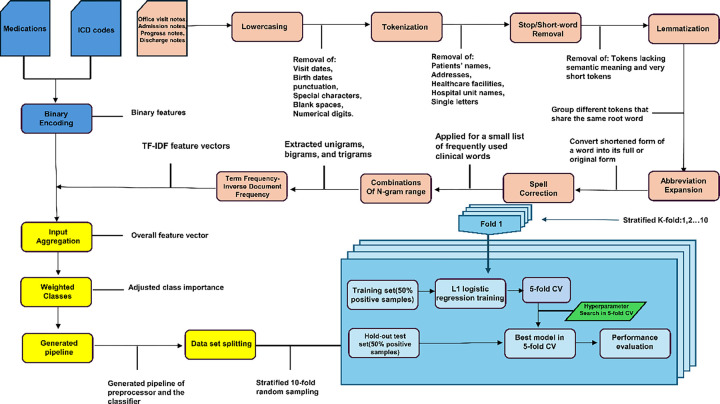
Method flowchart.

The entire method is depicted in Figure 2. Features included as input to the model included 9 groups of medications, 6 groups of ICD codes, and text features.

#### ICD code groupings:

ICD groupings and medications were defined a priori by three neurologists (MBW, SZ, SM), including: “Alzheimer’s disease” – ICD-10 F00, G30.0, G30.1, G30.8, G30.9 and ICD-9 290.0, 290.2x, 290.3, 331.0; “Vascular Dementia” – ICD-10 F01.X and ICD-9 290.4X; “Lewy Body Dementia” – ICD-10 G31.83 and ICD-9 331.82; “Frontotemporal Dementia” – ICD-10 G31.0, G31.01, G31.09 and ICD-9 311.11, 331.19; “Unspecified Dementias” – ICD-10 F02.8x, F03.9x and ICD-9 294.1x, 294.2x; “Mild Cognitive Impairment” ICD-10 F06.7 and ICD-9 331.83. One day before and after the visit was considered for assignment of an ICD code to account for prior or delayed data entry.

#### Medications:

Aricept, donepezil, Exelon, rivastigmine, memantine, Namenda, Namzaric, Razadyne, and galantamine ^21^.

#### Text features:

Keywords, phrases, and word patterns were extracted from notes. We converted the text in each note to lowercase, removed stop words and special characters, and applied lemmatization. Subsequently, we extracted unique words from each note. We also extracted unique bigrams (two consecutive words) and trigrams (three consecutive words) to identify potentially discriminative features. For each note, we created a vector representation of the information within the note using Term Frequency-Inverse Document Frequency (TF-IDF) weighting ^22,23^. TF-IDF quantifies the importance of a word within a note, relative to its prevalence across a collection of notes. Specifically, we created vectors containing TF-IDF values for each of the candidate features identified above. TF-IDF features were assembled into a single overall vector which serves as input to the classification model.

### Classification model training and testing

We trained a logistic regression model to assign a probability to each note, representing the likelihood of the clinical note indicating MCI/ADRD. To deal with imbalanced numbers of positive and negative subsamples, we used class weights inversely proportional to subsample size. LASSO regularization was employed for automated feature selection, with the relative importance of each feature assessed based on the magnitude of the resulting regression coefficients. To evaluate the performance of our model and assess generalizability, we implemented 5-fold stratified nested cross-validation. Hyperparameter optimization was conducted using internal cross-validation. To compare the informativeness of different data types, we trained models with the following input combinations: (1) clinical notes, ICDs, and medications combined, (2) clinical notes only; (3) ICDs only; and (4) medications only. Feature importance analysis was performed to determine which variables had the most significant impact on the model’s predictions.

### Model performance metrics

Model performance was evaluated using accuracy, precision, recall, specificity, F1-score, area under the AUROC, and area under the AUPRC ^24^. For each metric, we present micro-average performance metrics for positive and negative diagnoses of MCI/ADRD. We conducted 1000 iterations of bootstrapping to obtain the 95% confidence intervals (CI). Additionally, confusion matrices for various training and testing datasets further illustrate the model’s performance across different data splits. Error analysis was conducted to identify the primary sources of misclassification.

### Generalizability experiments

To evaluate the model’s generalizability across institutions and to enhance robustness by incorporating data from both, we conducted five experiments: 1) MGH as Training Set, BIDMC as Testing Set: We trained the model exclusively with data from MGH and tested the model on data from BIDMC. 2) BIDMC as Training Set, MGH as Testing Set: We trained the model exclusively with BIDMC and tested the model on MGH data. 3) MGH+ BIDMC Training Set, MGH+ BIDMC Testing Set: Training data came from both MGH and BIDMC, as did testing data. 4) MGH+ BIDMC Training Set, MGH Testing Set: Training data came from both MGH and BIDMC, testing data from MGH only. 5) MGH+ BIDMC Training Set, BIDMC Testing Set: Training data from both MGH and BIDMC, testing data from BIDMC only.

### Performance in unselected / random EHR samples

The sample utilized for training and testing is, by construction, enriched for “positive” cases, i.e. there are more MED+/ICD+, MED+/ICD−, and MED−/ICD+, and fewer MED−/ICD− cases than would be present in a random sample. Thus, although the overall error rate and other overall performance statistics calculated for our cohort are “biased”, i.e. they do not represent the performance that we would expect in a general, unselected hospital population. To obtain an unbiased estimate of model performance, we first estimated the error rates within each of the 4 groups $\left(P_{e}++,P_{e}+-,P_{e}-+,P_{e}--\right)$, then combined these with estimates of the prevalences of the 4 groups in the general hospital population to obtain an estimate of the unbiased error rate. Specifically, obtained estimates of the prevalences of each group (p++,p+-,p-+,p--) by sampling 500 patients randomly from BIDMC and 500 from randomly from MGH, and calculated the proportions falling within each of the 4 groups. The prevalences and error rates are then combined to give an overall expected error P[E] rate using the following formula:

$$P[E]=\left(P_{e}++\times p++\right)+\left(P_{e}+-\times p+-\right)+\left(P_{e}-+\times p-+\right)+\left(P_{e}--\times p--\right)$$

## Results

### Patient population

Figure 1 presents the CONSORT diagram illustrating the cohort selection process. The MGH cohort comprised 2,058 patients with 3,332 visits, while the BIDMC cohort included 1,819 patients with 3,479 visits. A total of 112 cases, accounting for 765 visits, were excluded due to uncertain manual annotations. After categorizing patients into four sampling groups, the final counts were as follows: 3,626 patients with 5,612 visits. Specifically, the ‘MED+ ICD+’ group had 133 patients with 1,751 visits; the ‘MED+ ICD−’ group included 466 patients with 481 visits; the ‘MED− ICD+’ group consisted of 214 patients with 214 visits; and the ‘MED− ICD−’ group contained 2,813 patients with 3,166 visits. In the subgroup analysis, the ‘MED+ ICD+’ group had 121 ADRD/MCI-positive patients with 1,643 visits and 12 MCI/ADRD-negative patients with 108 visits. The ‘MED+ ICD−’ group included 338 ADRD/MCI-positive patients with 353 visits and 121 MCI/ADRD-negative patients with 128 visits. The ‘MED− ICD+’ subgroup comprised 125 ADRD/MCI-positive patients with 125 visits and 89 MCI/ADRD-negative patients with 89 visits. The largest group, ‘MED− ICD−’, included 522 ADRD/MCI-positive patients with 592 visits and 2,291 MCI/ADRD-negative patients with 2,574 visits. The cohort was selected to ensure sufficient patients with MCI/ADRD diagnoses for model training. Consequently, the final MGH and BIDMC cohort included 1,106 (30.5%) MCI/ADRD-positive patients from 1,634 visits and 2,587 MCI/ADRD-negative patients from 3,978 visits.

Table 1 summarizes the baseline characteristics of the cohort. The average age was 67.6 years, with 47% of patients being male and 53% female. Racial distribution included 16.0% Black or African American, 1.5% Asian, 70.5% White, and 11.94% categorized as ‘Other’. The MCI/ADRD diagnosis rate was 30.5%. Among the four sampling groups, the ‘MED+ ICD+’ group had 10.94%, the ‘MED+ ICD−’ group had 30.56%, the ‘MED− ICD+’ group had 11.3%, and the ‘MED− ICD−’ group had 47.2%.

### Model performance

Performance results for predicting MCI/ADRD chart diagnoses are presented in Table 2, which varies the model inputs, and Table 3, which varies the training and testing cohorts. Table 2 presents the average performance for logistic regression models using ICD Only, Med Only, Note Only, and ICD+MED+Note inputs in the MGH+BIDMC training sets and MGH+BIDMC testing sets. The findings indicate a clear pattern in the performance of logistic regression models based on different input data types. Models that incorporate textual note data, either alone or in combination with ICD codes and medication data, consistently outperform models using only ICD codes or only medication data across all performance metrics, with an accuracy of 0.89, specificity of 0.90, AUROC of 0.95, and AUPRC of 0.95. In Table 3, the highest performance was observed when the MGH+ BIDMC training set was tested on the MGH set, achieving an AUROC of 0.98, an AUPRC of 0.98, an accuracy of 0.93, a specificity of 0.96, a precision of 0.96, an F1 score of 0.93 and a recall of 0.91.

**Figure 3:**
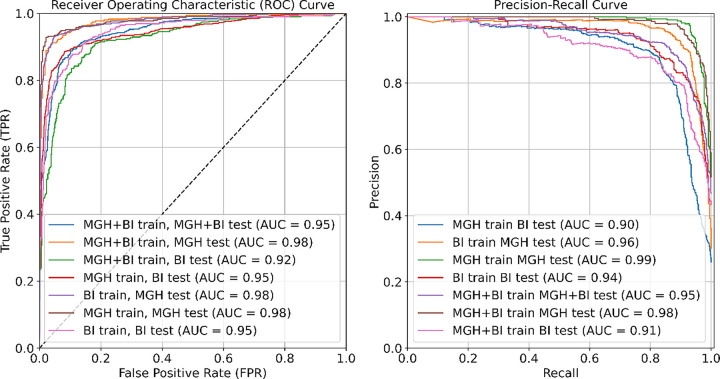
Left panel: Comparative analysis of all training/testing approaches using Receiver Operating Characteristic (ROC) curves. Right panel: Comparative analysis of all training/testing approaches using Precision-Recall curves.

Figure 3 provides a comparative analysis of various training and testing approaches using ROC curves (left panel) and Precision-Recall (PR) curves (right panel). The highest ROC observed is 0.99 for the MGH training and MGH testing set. The Precision-Recall curves demonstrate the trade-off between precision and recall, with the highest Precision-Recall AUC being 0.98 for multiple model configurations, including MGH training tested on the MGH set and MGH+BIDMC training tested on the MGH set.

**Figure 4:**
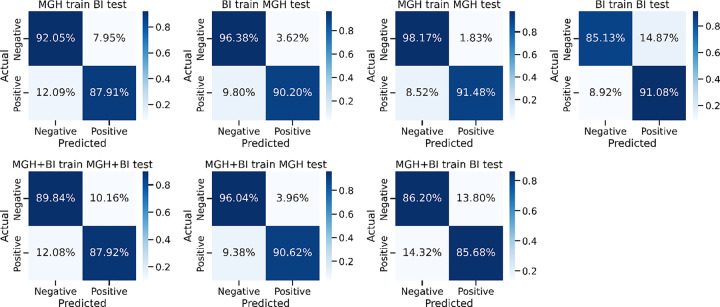
Comparative analysis of all training/testing approaches using confusion matrix.

Figure 4 shows the confusion matrices representing various training/testing experiments in the context of predicting MCI/ADRD. The columns correspond to predicted MCI/ADRD status, while rows represent the ground truth classification based on chart review. The model trained and tested on MGH data shows the highest accuracy, with 98.17% for negative and 91.48% for positive predictions. Conversely, models trained on one dataset and tested on another exhibit lower performance, particularly in positive predictions. Combining both datasets for training (MGH+BI) and testing on the combined or individual datasets yields intermediate performance.

**Figure 5:**
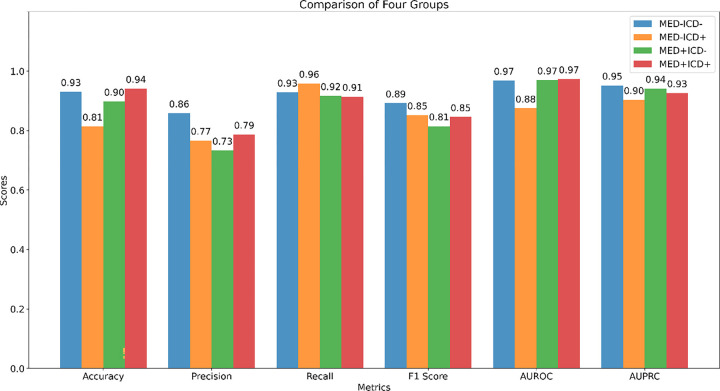
Performance Evaluation of Different Subgroups Across Multiple Metrics.

Figure 5 compares the performance of the model across the four sampling groups, ‘MED−ICD−’, ‘MED−ICD+’, ‘MED+ICD−’, and ‘MED+ICD+’. Performance is consistently higher for all metrics except recall in the patients with congruent ICD and medication information (‘MED−ICD−’, ‘MED+ICD+’ subgroups) across most metrics, and lower for patients with ‘mixed’ information (‘MED+ICD−’, ‘MED−ICD+’). In terms of accuracy, the ‘MED+ICD+’ group performed best (0.94) and ‘MED−ICD+’ performed worst (0.81). Precision was highest for ‘MED−ICD−’ (0.86) and lowest for ‘MED+ICD−’ (0.73). Recall was best for ‘MED−ICD+’ (0.96) with little difference among the other groups. The F1 Score was highest for ‘MED−ICD−’ (0.89) and lowest for ‘MED+ICD−’ (0.81). AUROC was the same for ‘MED−ICD−’, ‘MED+ICD−’, and ‘MED+ICD+’ (0.97), while ‘MED−ICD+’ was the lowest (0.88). AUPRC was highest for ‘MED−ICD−’ (0.95) and lowest for ‘MED−ICD+’ (0.90).

**Figure 6:**
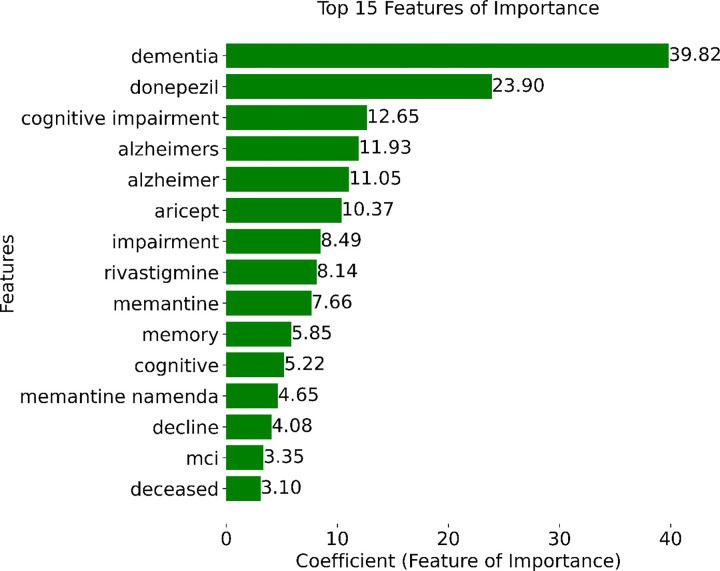
Top 15 important features based on model coefficients.

Figure 6 shows the coefficient values of the top 15 features selected during model training. Notably, the presence of the word “dementia” in a note emerged as the most informative feature, followed closely by the prescription of MCI/ADRD-related medications, including donepezil, aricept, rivastigmine, and memantine. Other top-15 MCI/ADRD-related keywords included “cognitive impairment”, “Alzheimer”, “MCI”, “memory”, “cognitive”, and “decline”.

### Performance in unselected / random EHR samples

We calculated the expected error rate in a general hospital population following the procedure described in the Methods section. In the random sample selected (N = 1000, 500 from MGH, 500 from BIDMC), the proportion (and numbers) falling within each of the 4 groups were: MED+ ICD+, $P_{e}++=.1\%\;(n=1)$; MED+ICD−, $P_{e}+-.4\%\;(n=4)$; MED− ICD+, $P_{e}-+=0.3\%\;(n=3)$; MED−ICD−, $P_{e}--=0.2\%\;(n=2)$. Combining these with the error rates in each group (p++,p+-,p-+,p--), we obtain as an estimate of the overall (unbiased) error rate:

$$P[E]=\left(P_{e}++\times p++\right)+\left(P_{e}+-\times p+-\right)+\left(P_{e}-+\times p-+\right)+\left(P_{e}--\times p--\right)\;\;=(0.06\times 0.001)+(0.1\times 0.004)+(0.19\times 0.003)+(0.07\times 0.002)\;\;=0.00006+0.0004+0.00057+0.00014=0.00117\approx 0.12\%$$

The overall error rate in the general hospital population is estimated to be 0.12%, i.e. accuracy of 99.88%.

### Error analysis

We conducted a manual review of cases to gain qualitative insights into reasons for model errors. False positives arose primarily from clinical notes describing symptoms resembling MCI/ADRD, such as memory loss and cognitive decline attributable to alternative causes such as depression and anxiety. Conversely, false negatives arose primarily from notes from specialists seeing patients with MCI/ADRD for specialized care in other areas of medicine, such as nephrology or gynecology, who commented sparsely on issues related to the MCI/ADRD diagnosis in their notes.

## Discussion

Our machine learning-based automated EHR phenotyping model accurately identifies patients diagnosed with MCI/ADRD using unstructured clinical notes, ICD codes, and MCI/ADRD medications. Models incorporating textual notes, either alone or combined with ICD codes and medication data, consistently outperformed those relying solely on ICD codes or medication data. The ICD+MED+Note model achieved the highest performance, underscoring the importance of integrating diverse data sources for enhanced accuracy and reliability. As shown in Table 2, models using only ICD codes exhibit lower specificity (i.e., higher false positive rate). In contrast, models based solely on clinical notes demonstrate superior performance, with notable differences in AUROC (0.94 vs. 0.54), AUPRC (0.69 vs. 0.95), and F1-score (0.78 vs. 0.60).

Our analysis revealed an important finding that the information in clinical notes leads to much better performance than using ICD codes alone ^25,26^. For example, the ICDs may be triggered by a broad range of cognitive symptoms that are not necessarily due to MCI or dementia. This is more likely in the older population where other disease conditions are common, including medication side effects or interactions, depression /mood difficulties^27^, hypothyroidism, substance use (such as alcohol and marijuana), and sleep difficulties^28^. The accurate diagnosis of MCI or dementia requires comprehensive testing^29–31^, including cognitive testing, physical examination, and often neuroimaging ^32^. The clinical notes often contain more detailed descriptions and therefore more information about the ground truth.

The performance indicates good generalizability across sites. Nevertheless, there are notable site differences. Performance on MGH test data was better compared to performance on BIDMC test data, regardless of the source (BIDMC or MGH) of the training data, suggesting that the MGH dataset may present fewer complexities or challenges. Conversely, adding data from BIDMC to the training dataset resulted in a slight decline in performance on tests conducted at BIDMC. Specifically, the AUROC decreased from 0.98 to 0.92, and the AUPRC decreased from 0.98 to 0.91. These changes represent a minimal impact on the model’s performance. The alignment of the features identified for retention within the model with existing medical knowledge and their consistency with an established understanding of dementia and MCI/ADRD medications suggests that the model has learned a reasonable, interpretable pattern. In the future, we hope to apply this model to identify MCI/ADRD patients from electronic health records at scale, thus creating opportunities for large-scale EHR-based studies.

A strength of our approach is the use of data across two health networks (MGH and BIDMC); most published studies focus on single-site data^25,33–35^. This multi-site comparison allowed for a broader validation of our model, showing consistency in performance metrics such as accuracy, specificity, and AUC across different institutional datasets. Our model achieved high AUROC (0.98), demonstrating robust discrimination across testing scenarios. We also emphasize the importance of the highly observed AUPRC, particularly as it relates to the clinical relevance in contexts where class imbalance is pronounced. The high AUPRC indicates that our model effectively identifies “rare” events, such as ADRD/MCI diagnoses, from large healthcare datasets. This strong performance in correctly identifying true positive cases is crucial for accurately diagnosing fewer common conditions with significant clinical implications.

Additionally, our study included a larger patient cohort than those typically reported ^25,33–35^, which enhances the generalizability of our findings. References to other studies comparing site performance are scarce, making our contributions significant for future multicentric studies. The testing performance was generally better on MGH data than on BIDMC data, regardless of the site of origin of the training data. This suggests that there may be a larger proportion of difficult or ambiguous cases in the BIDMC test set. Nevertheless, test performance was excellent for both sites, suggesting generalizability across notes written within different medical institutions.

Our study has important limitations. While our experiments included two medical centers, these are located in the same geographic region (Boston, United States), and may thus not be representative of other US and non-US populations. Thus, future studies that utilize our model across different hospitals and EHR systems should check for performance biases that might arise due to different demographics, larger sample sizes, bias in data collection, and EHR data stored formats. An additional limitation is that our model does not identify specific subtypes of ADRD and provides no information about the severity of ADRD or MCI. Incorporating large language models (LLMs) like GPT may enhance the feature extraction and interpretation of clinical notes by handling complex medical language and context-specific nuances more effectively – an approach we did not use. Overall, this study represents an important step towards unlocking the vast potential of EHR data to advance our understanding of mild cognitive impairment and dementia and enables various downstream studies.

In conclusion, our model combining the clinical notes, ICD codes, and medications from the EHR system provides accurate MCI/ADRD phenotyping. In the future, this work will enable important downstream large-scale analyses to understand various aspects of MCI/ADRD.

## Figures and Tables

**Table 1:** Cohort characteristics.

Characteristic	Value( *N* =3,626)
**Age** ^(a)^ **(years, mean (SD))**	67.6 (16.7)
Sex, n (%)	
Male	1704 (47%)
Female	1922 (53%)
**Race, n (%)**	
Black or African American	580 (16.0%)
Asian	55 (1.5%)
White	2558 (70.5%)
Other ^(b)^	433 (11.94%)
**MCI/ADRD diagnosis, n (%)**	1106 (30.5%)
MED+ICD+ group	121 (10.94%)
MED+ICD- group	338 (30.56%)
MED-ICD+ group	125 (11.3%)
MED-ICD- group	522 (47.2%)

(a) Age at baseline for the first visit in the study period.

(b) ‘Other’ includes ‘unknown’, ‘declined to answer’, ‘American Indian or Alaska Native’ and ‘Native Hawaiian or other Pacific Islander’

**Table 2:** Average performance and [95% confidence intervals] for logistic regression models using ICD Only, Med Only, Note Only, and ICD+MED+Note in the MGH+BIDMC training sets and MGH+BIDMC testing sets

Model Input	Accuracy	Specificity	F1-score	Recall	Precision	AUROC	AUPRC
ICD Only	0.54 [0.52–0.55]	0.37 [0.35–0.38]	0.60 [0.59–0.62]	0.7 [0.69–0.72]	0.53 [0.51–0.55]	0.54 [0.52–0.55]	0.69 [0.68–0.70]
Med Only	0.56 [0.55–0.58]	0.43 [0.41–0.45]	0.61 [0.60–0.63]	0.69 [0.68–0.71]	0.55 [0.53–0.57]	0.56 [0.55–0.58]	0.70 [0.69–0.71]
Note Only	0.88 [0.87–0.91]	0.90 [0.89–0.93]	0.88 [0.86–0.90]	0.87 [0.85–0.88]	0.88 [0.87–0.91]	0.94 [0.93–0.95]	0.94 [0.93–0.95]
ICD+MED+Note	0.89 [0.88–0.90]	0.90 [0.89–0.92]	0.88 [0.87–0.90]	0.88 [0.86–0.90]	0.88 [0.88–0.91]	0.95 [0.94–0.96]	0.95 [0.94–0.96]

AUROC: Area Under the Receiver Operating Characteristic curve, shows model’s ability to distinguish between classes.

AUPRC: Area Under the Precision-Recall Curve, summarizes the precision and recall across different thresholds.

Inputs: ICD Only: Models using only International Classification of Diseases codes. Med Only: Models using only medication data.

Note Only: Models using only textual note data. ICD+MED+Note: Models combining ICD codes, medication data, and textual note data.

Data Sets: MGH+ BIDMC: Data derived from Massachusetts General Hospital and Beth Israel Deaconess Medical Center.

**Table 3:** Average performance and [95% confidence intervals] for logistic regression model using all features in the different testing sets

Training set	Testing set	Accuracy	Specificity	F1-score	Recall	Precision	AUROC	AUPRC
MGH+BIDMC	BIDMC	0.86 [0.84–0.87]	0.86 [0.83–0.87]	0.84 [0.82–0.86]	0.85 [0.83–0.88]	0.82 [0.80–0.84]	0.92 [0.91–0.94]	0.91 [0.90–0.93]
MGH	BIDMC	0.90 [0.89–0.92]	0.92 [0.91–0.93]	0.83 [0.81–0.85]	0.88 [0.86–0.90]	0.79 [0.77–0.83]	0.95 [0.93–0.96]	0.90 [0.89–0.92]
BIDMC	MGH	0.94 [0.94–0.95]	0.96 [0.96–0.97]	0.91 [0.89–0.92]	0.90 [0.88–0.91]	0.91 [0.91–0.93]	0.98 [0.97–0.98]	0.98 [0.97–0.98]
MGH+BIDMC	MGH	0.93 [0.92–0.95]	0.96 [0.95–0.98]	0.93 [0.92–0.95]	0.91 [0.88–0.92]	0.96 [0.94–0.99]	0.98 [0.97–0.99]	0.98 [0.98–0.99]
MGH+BIDMC	MGH+BIDMC	0.89 [0.88–0.90]	0.90 [0.89–0.92]	0.88 [0.87–0.90]	0.88 [0.86–0.90]	0.88 [0.88–0.91]	0.95 [0.94–0.96]	0.95 [0.94–0.96]

The bootstrapping results in 95% confidence intervals are in parenthesis. ACC – accuracy, Spec – specificity, AP – average precision, AUROC – Area under the receiver operating characteristic curve, AUPRC – area under the precision-recall curve. Data Sets: MGH: Data derived from Massachusetts General Hospital. BI: Data derived from Beth Israel Deaconess Medical Center. MGH+ BIDMC: Data derived from Massachusetts General Hospital and Beth Israel Deaconess Medical Center.

## Data Availability

All data and computer code used to produce the figures and tables will be available at the time of publication here: (https://github.com/rockey1006/Automated_ADRD-MCI) and here (https://bdsp.io/projects/jpCoV5N1MsB9a5QsgprQ/).
